# Inhibition of glioblastoma dispersal by the MEK inhibitor PD0325901

**DOI:** 10.1186/s12885-017-3107-x

**Published:** 2017-02-10

**Authors:** Stephen Shannon, Dongxuan Jia, Ildiko Entersz, Paul Beelen, Miao Yu, Christian Carcione, Jonathan Carcione, Aria Mahtabfar, Connan Vaca, Michael Weaver, David Shreiber, Jeffrey D. Zahn, Liping Liu, Hao Lin, Ramsey A. Foty

**Affiliations:** 1 Department of Surgery-Rutgers Robert Wood Johnson Medical School, Clinical Academic Building, 125 Paterson Street, New Brunswick, NJ 08901 USA; 2 Rutgers-Department of Biomedical Engineering, 599 Taylor Road, Piscataway, NJ 08854 USA; 3 Rutgers-Department of Mechanical and Aerospace Engineering, 98 Brett Rd, Piscataway Township, NJ 08854 USA; 4 Rutgers-Department of Mathematics, 110 Frelinghuysen Rd, Piscataway, NJ 08854 USA

**Keywords:** Glioblastoma, Dispersal velocity, MEK inhibitor, 3D spheroids, Fibronectin matrix

## Abstract

**Background:**

Dispersal of glioblastoma (GBM) cells leads to recurrence and poor prognosis. Accordingly, molecular pathways involved in dispersal are potential therapeutic targets. The mitogen activated protein kinase/extracellular signal regulated kinase (MAPK/ERK) pathway is commonly dysregulated in GBM, and targeting this pathway with MEK inhibitors has proven effective in controlling tumor growth. Since this pathway also regulates ECM remodeling and actin organization − processes crucial to cell adhesion, substrate attachment, and cell motility – the aim of this study was to determine whether inhibiting this pathway could also impede dispersal.

**Methods:**

A variety of methods were used to quantify the effects of the MEK inhibitor, PD0325901, on potential regulators of dispersal. Cohesion, stiffness and viscosity were quantified using a method based on ellipsoid relaxation after removal of a deforming external force. Attachment strength, cell motility, spheroid dispersal velocity, and 3D growth rate were quantified using previously described methods.

**Results:**

We show that PD0325901 significantly increases aggregate cohesion, stiffness, and viscosity but only when tumor cells have access to high concentrations of fibronectin. Treatment also results in reorganization of actin from cortical into stress fibers, in both 2D and 3D culture. Moreover, drug treatment localized pFAK at sites of cell-substratum adhesion. Collectively, these changes resulted in increased strength of substrate attachment and decreased motility, a decrease in aggregate dispersal velocity, and in a marked decrease in growth rate of both 2D and 3D cultures.

**Conclusions:**

Inhibition of the MAPK/ERK pathway by PD0325901 may be an effective therapy for reducing dispersal and growth of GBM cells.

**Electronic supplementary material:**

The online version of this article (doi:10.1186/s12885-017-3107-x) contains supplementary material, which is available to authorized users.

## Background

Early and continuing dispersal of tumor cells from the primary mass renders GBM refractory to complete surgical excision or targeted chemotherapy and directly leads to recurrence and dismal prognosis. Strategies aimed at containing the primary or recurrent tumor could significantly improve targeted delivery of chemotherapeutic agents and increase the likelihood of total surgical resection. To disperse, cells must first detach from the primary mass, a process that likely involves mechanisms that decrease cohesion between tumor cells [1]. Cells must also attach to substrates at strengths that optimize their motility and secrete factors to facilitate their interaction with parenchyma [2, 3]. In addition, tumor cells must also become relatively compliant so as to deform and “squeeze” through pores in a meshwork of ECM components [4], and in the case of GBM, astrocytes within the normal brain parenchyma. Accordingly, strategies aimed at preventing tumor cell detachment, limiting motility, and inhibiting changes in compliance offer an effective approach to reduce dispersal. Ideally, such strategies should employ pharmacological agents that can cross the blood–brain barrier and that specifically target molecular pathways involved in mediating cohesion, adhesion, and compliance.

Cadherins, integrins and the extracellular matrix (ECM) are potential therapeutic targets, and various studies have identified drugs that can modulate their expression or function. For example, gamma-linolenic acid (GLA) up-regulates E-cadherin expression and inhibits invasion of lung, colon, breast, melanoma, and liver cancer [5]. Invasion suppression here was likely due to an increase in the strength of intercellular cohesion mediated by up-regulation of E-cadherin. 5-aza-deoxycitidine (5 AC) has also been shown to effectively inhibit invasion by up-regulating E-cadherin expression [6]. Because the down-regulation of E-cadherin is often associated with up-regulation of N-cadherin during epithelial-mesenchymal transition, drugs that can block N-cadherin expression have also been shown to be effective in blocking invasion. Biflorin, a novel o-naphtoquinone, has been shown to inhibit expression of N-cadherin and to block invasion of breast cancer cells [7]. Such drugs could be of potential benefit for glioblastoma given the correlation between increased N-cadherin expression in high-grade gliomas and tissue invasion [8]. Various integrins, including αvβ3 and αvβ5 have also been targets of anticancer therapy. Cilengitide, a cyclic pentapeptide, is a specific inhibitor of these integrins and has been shown to have anti-invasive activity in various glioma models [9]. Given the complexity and heterogeneity of the ECM, and the likelihood that glioma cells tune their integrin receptor fingerprint to match the local ECM microenvironment, drugs that modulate the ECM may prove effective in reducing dispersal. Many of these drugs, including various corticosteroids, target the ECM as a by-product of the drugs’ principal actions. Consequently, this activity may in part be beneficial to the drugs’ disease-modifying properties [10]. An example of such a drug is Dexamethasone (Dex). Dex is currently used to treat brain tumor-related edema associated with mass effect from Glioblastoma [11]. A by-product of the effects of Dex in glioblastoma is its ability to restore fibronectin matrix assembly (FNMA) and decrease detachment of tumor cells from cultured 3D spheroids [1]. However, due to the relatively high doses required, Dex has many side-effects, often limiting its long-term use. Identification of other drugs that can have similar effects but more specifically target pathways involved in modulating integrins and the ECM could be of therapeutic value.

The MAPK/ERK pathway has been identified as a commonly dysregulated pathway in several cancers, most notably in melanoma. Combined targeting of this pathway can have a synergistic effect in controlling tumor growth [12]. Clinical trials using various MEK inhibitors, such as trametinib [13, 14], cobimetinib [15] and CI 1040 (PD184352) [16] have been shown to shrink some melanomas, specifically those with BRAF mutations. The MEK inhibitor PD0325901 has also demonstrated efficacy in melanoma cell lines independent of BRAF status [17]. Experimental models have demonstrated in vitro and in vivo efficacy of PD0325901 in controlling tumor growth in animal models of GBM [18], although studies have identified possible issues with limited access through the blood–brain barrier [19]. To our knowledge, there is only one ongoing phase-2 trial testing the effects of PD0325901 on tumor growth in patients with neurofibromatosis type −1 (NF1) or plexiform neurofibromas [20] NCT02096471), and none testing efficacy in GBM. The majority of these studies have focused mainly on inhibition of growth and on activation of apoptosis. Inasmuch as MEK inhibitors target pathways that can also influence actin organization and remodeling of the ECM, we asked whether PD0325901 could also serve to impact mechanisms that regulate dispersal of primary human GBM cells.

We first determined whether primary human GBM cells used in this study are sensitive to PD0325901. We then assessed the effects of MEK inhibition on integrin activation *vis à vis* restoration of FNMA and actin organization in both 2D and 3D cultures. We also quantified the effects of PD0325901 on spheroid mechanical properties including cohesion, stiffness and viscosity. We evaluated effects of PD0325901 in regulating the strength of cell-substrate adhesion, cell motility, dispersal of tumor cells from spheroids, and in an ex vivo dispersal assay. Finally, we determined whether PD0325901 could also influence the growth rate of both 2D and 3D cultures of GBM.

## Methods

### Cell lines, maintenance, treatment, and generation of 3D spheroids

Four human primary glioblastoma cell lines (GBM-1, GBM-2, GBM-3 and GBM-4) were previously isolated and characterized [21]. Samples were examined by a neuropathologist and stained for several markers to confirm their designation as human GBM. Microscopically, all lines were described as astrocytic neoplasms with moderate to high pleiomorphism, vascular endothelial hyperplasia, with areas of abundant necrosis. Lines are all GFAP positive. GBM-1 and GBM-4 exhibit PTEN loss and all lines appear to express p-AKT. All lines express Nestin and BMI-1, both markers of undifferentiated cells. Collectively, pathologic and molecular analysis confirms highly undifferentiated grade IV glioma/glioblastoma. Cells were maintained in Eagles’ Minimal Essential Medium (EMEM)/10% fetal calf serum (FCS) and antibiotics/antimycotics. They were sub-cultured using standard protocols and used at 3^rd^ to 6^th^ passage. Normal human astrocytes (NHA) were purchased from Lonza (Allendale, NJ) and maintained in AGM™ Astrocyte Growth Medium as recommended by the manufacturer. Where required, cells were treated with PD 0325901, a powerful inhibitor of ERK1/2 phosphorylation, at a final concentration of 1 μM w/v DMSO for 24 h prior to assay. Spheroids were generated as previously described [1].

### Immunoblot and immunofluorescence assays

To confirm that PD0325901 inhibited ERK1/2 phosphorylation, cells were treated with either dimethyl sulfoxide (DMSO, vehicle control) or 1 μM PD0325901 overnight under standard tissue culture conditions. Twenty μg of protein was separated by SDS-PAGE under reducing conditions. Gels were blotted to PVDF and probed with anti-phospho P44/42 MAPK or P44/42 MAPK antibodies (Cell Signaling Technologies, Danvers, MA) and appropriate HRP-conjugated secondary antibodies. Blots were developed using Amersham ECL Prime Western Blotting Detection reagent (GE Healthcare Life Sciences, Pittsburgh, PA) and a C-Digit Blot Scanner (Li-COR, Lincoln, NE). Assessment of FNMA, phospho-FAK and actin expression by GBM cells in conventional 2D culture was performed as previously described [1]. For assessment of actin organization in 3D spheroids, aggregates of GBM cells were fixed and permeabilized with 4% paraformaldehyde/0.5% Triton X-100 and incubated in 6nM rhodamine-phalloidin for 30 min. Aggregates were washed 4x with PBS, mounted onto slides, and imaged using a Zeiss AxioImager Z1 spinning disc confocal microscope attached to a Photometrics Evolve 512 EMCCD camera with Metamorph Premier imaging software.

### Measurement of aggregate cohesion and viscoelasticity

Aggregate cohesion was measured by tissue surface tensiometry (TST). TST employs a custom-built instrument to compress spherical cellular aggregates between poly-HEMA coated parallel plates to which they cannot adhere. Measurements of aggregate geometry and resistance to the applied force are then applied to the Young-Laplace equation to calculate aggregate surface tension. The method has been described in detail [1, 22–24]. TST measurements are only valid when tissues behave like liquid systems [22–24]. Accordingly, the calculated surface tension of a liquid aggregate, when subjected to two successive compressions (σ_1_ and σ_2_), the second greater than the first, will remain constant. In such aggregates the ratio of σ_2_/σ_1_ will approach 1 and will be less than the ratio of the force applied at each successive compression (F_2_/F_1_). The surface tension of liquid aggregates will also be independent of aggregate size. Only measurements in which surface tension is independent of the applied force and size were used to calculate average σ for each cell line.

For measurement of viscoelasticity, aggregates ranging in size from 200-400 μm were loaded into the tensiometer and subjected to a compressive force for 30 s, whereupon the force was removed and aggregates were allowed to relax for 2 min. A high-speed camera captured 12 frames/s and the shape of the relaxing aggregates was extracted and analyzed using an in-house edge detection and analysis algorithm. Mechanical parameters were extracted from the shape dynamics with a continuum-based model which includes a Kelvin-Voigt bulk enclosed in a stressed surface. This advanced model is different than the simple spring-dashpot or compartmental models previously described [25]. Analysis of the relaxation dynamics was greatly facilitated by a closed-form, analytical solution that we derived. Details of the theory and the data analysis method, as well as preliminary data validating our approach are presented in Additional file 1.

### Measurement of shear-flow induced detachment

Cell-ECM attachment was measured by subjecting adhering cells to flow-induced shear stress as previously described [1]. Briefly, DMSO or PD0325901-treated GBM cells were plated at a concentration of 5x10^4^ cells/ml onto 6-well polyethylene terephthalate cell culture inserts (Franklin Lakes, NJ) for 2 h and were then inverted into complete medium and incubated overnight. Inserts were then loaded into custom-designed flow chambers and subjected to 30 dynes/cm of shear stress for 3 h, whereupon inserts were washed in PBS and immersed in SYTO 16 green fluorescent nucleic acid stain (Life Technologies, Carlsbad, CA). Cells seeded onto inserts but not subjected to flow were used as growth rate controls. A Nikon Eclipse epifluorescence microscope was used to capture nine low magnification fields/insert and nuclei were counted in ImageJ. The average number of attached cells was then expressed as a percentage of the no-flow controls.

### Measurement of cell motility

GBM cell motility was measured using a fluorescence bead phagokinetic assay [26] as previously described [1]. Briefly, wells of a six-well dish were coated with poly-D-lysine, whereupon 1 μM diameter fluorescent polystyrene microspheres (ThermoFisher Scientific, Grand Island, NY), adjusted to a concentration of 0.018% v/v in PBS were added and allowed to adhere to the poly-lysine for 2 h. Cells were plated in complete tissue culture medium (TCM) at a cell/area density of 4 cells/mm^2^. Experiments were performed either in DMSO or in 1 μm PD0325901. Experiments were also performed with PD0325901-treated cells incubated in hFn 7.1, a mouse monoclonal anti-human fibronectin antibody, or with non-specific mouse IgG. Motile cells phagocytose beads as they move leaving behind non-fluorescent tracks. Cleared area was quantified in ImageJ.

### Measurement of aggregate dispersal velocity

50–100 μm diameter aggregates of DMSO or PD0325901-treated GBM-1-4 were deposited into 12-well tissue culture plates containing 2mls of pre-warmed TCM. Plates were incubated for eight h. Images were captured for each aggregate every hour and diameter at each time point was measured. Dispersal velocity (DV) was represented by the slope as determined by linear regression analysis for change of diameter as a function of time. Only regression lines with r^2^ values of 0.95 and greater were used to calculate DV for each GBM line. Data were normalized with initial aggregate diameter. Twelve aggregates were used to generate an average DV for each GBM line.

### Measurement of z-axis dispersal distance by confocal microscopy

Dispersal of GBM cells through a NHA-seeded porous filter was measured as previously described [1]. Two-hundred μm thick, cross-linked polystyrene scaffolds (Alvetex, Reinnervate, Durham, UK) with tunnel diameters of 8–13 μm were seeded with 1x10^6^ NHA cells in 100 μL of tissue culture medium. After 60 min to allow NHA cells to adhere, scaffolds were placed in 12-well plates and incubated in 4mls of TCM for 48 h to permit incorporation of NHA cells throughout the scaffold. After 48 h, GBM cells that had been transfected with BacMam 2.0 GFPT (Life Technologies, Long Island, NY) were deposited onto each scaffold in a small volume of medium. Scaffolds were incubated for 48 h to allow time for tumor cells to infiltrate and disperse. To image dispersed cells, a Yokogawa CSU-X1 spinning disk confocal microscope with MetaMorph software was used to generate z-stacks of images taken at 1 μm intervals. Differential interference contrast microscopy was used to identify the z = 0 starting point for each z-stack. The z-axis position of each cell within each tissue-scaffold was scored. Within any given scaffold the mean average z-axis cell position from 5– 6 z-stacks was measured and recorded.

### Measurement of cell growth in conventional 2D culture and in 3D spheroids

For measurement of growth in conventional 2D cultures, cells were plated at a concentration of 5x10^4^ cells/ml in wells of a 6-well dish in complete medium. Total and live cell counts were performed once/day for 4 days using a BioRad TC10 automated cell counter. For measurement of growth rate by 3D spheroids, aggregates were generated using the hanging drop method [1]. Single aggregates were plated onto wells of an agarose-coated 6-well dish. Agarose prevented aggregates from adhering to the bottom of the dish. The area of each aggregate was measured once/day for nine days. Growth rate was determined by plotting aggregate area as a function of time. Regression analysis was performed to calculate growth rates of 3D spheroids [1].

## Results

### Effects of PD0325901 on FNMA, actin organization and pFAK localization in primary GBM cells

**Fig. 1:**
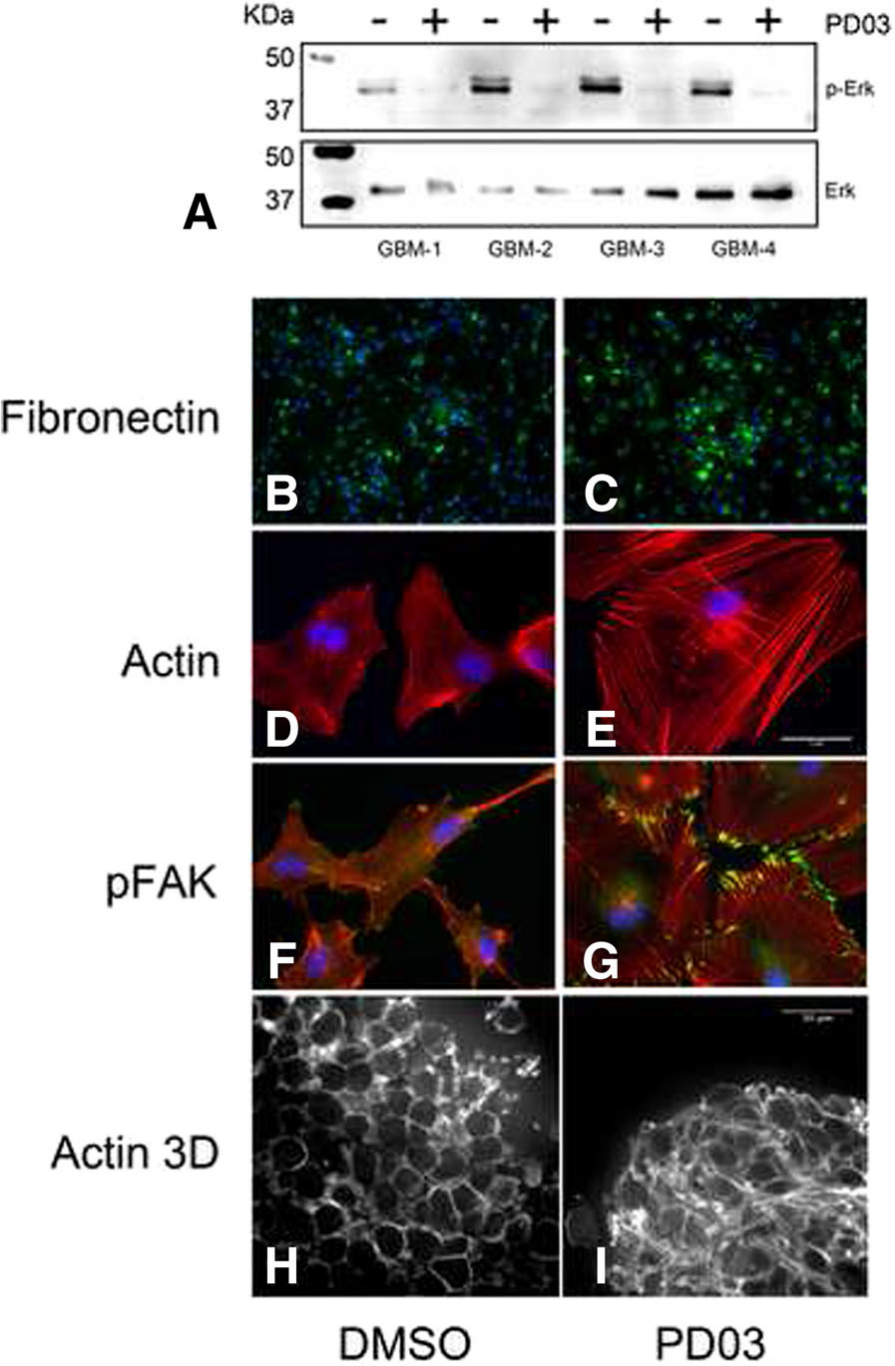
Effects of PD0325901 on FNMA, actin organization and pFAK localization in primary GBM cells. Immunoblot analysis for phosho-ERK and ERK in response to overnight treatment with 1 μm PD0325901 or DMSO as vehicle control. PD0325901 significantly inhibited phosphorylation of ERK (**a**). Representative immunofluorescence images of FNMA by GBM-3 cells treated either with DMSO (**b**) or PD0325901 (**c**). Fibronectin is depicted in green and DAPI (blue) was used as counterstain. PD0325901 did not appear to induce FNMA by GBM-3 cells. Rhodamine-phalloidin staining of actin in DMSO-treated (**d**) or PD0325901-treated GBM-3 cells (**e**). Note significant cell shape change and actin fiber organization. Scale bar in (**e**) is 5 μm. Triple stain for actin (*red*), p-FAK (*green*) and DAPI (*blue*) in DMSO-treated (**f**) and PD0325901-treated (**g**) GBM-3 cells. PD0325901 appears to induce the localization of p-FAK at sites of cell-ECM attachment. Thirty-micron thick z-stack of DMSO (**h**) and PD0325901-treated (**i**) collected by confocal microscopy of multicellular aggregates of GBM-3. Note marked change in actin organization from cortical to stress fibers. Scale bar in (**i**) is 30 μm

Studies have previously demonstrated a growth-inhibitory role for PD0325901 in GBM [20]. Here, we explore another potential role as a suppressor of GBM dispersal. We first confirmed that the primary lines used in this study are sensitive to drug treatment. Figure 1a shows that PD0325901 treatment down-regulates p-ERK, the downstream effector of MEK, in all 4 primary GBM cell lines. Unlike Dex, PD0325901 did not induce FNMA (Fig. 1c) relative to DMSO controls (Fig. 1b). Rather, treatment resulted in a remarkable change in cell shape, treated cells (Fig. 1e) becoming flatter and larger than those treated with DMSO (Fig. 1d). PD0325901 treatment also gave rise to the organization of actin into stress fibers when cells were grown as conventional 2D culture (Fig. 1d, e), and a shift in actin organization from cortical to stress fibers when cells were incubated as 3D hanging drops (Fig. 1h, i). Moreover, PD0325901 treatment resulted in the localization of p-FAK at sites of cell-ECM attachment (Fig. 1g). These results indicate that PD0325901 treatment activates mechanisms involved in regulating cell motility and mechanical properties of single cells or cellular aggregates.

### The effects of PD0325901 on spheroid mechanical properties are fibronectin dependent

**Fig. 2:**
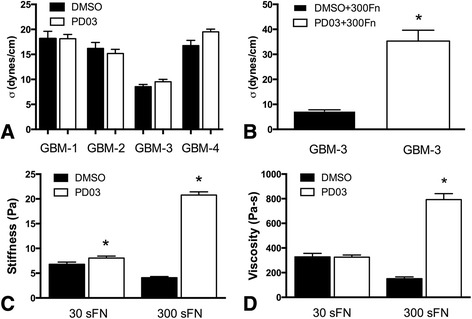
Assessment of aggregate cohesion, stiffness, and viscosity in response to PD0325901 treatment. Surface tension measurements for GBM-1-4 aggregates generated using standard TCM and treated with either DMSO or PD0325901. *n* = 20, pair-wise comparison by Student *t*-test, *p* > 0.05 (**a**). Surface tension measurements of GBM-3 aggregates generated in fibronectin-depleted medium supplemented with 300 μg/ml of human fibronectin. *n* = 20, pair-wise comparison by Student *t*-test, *p* < 0.0001 (**b**). Stiffness (**c**) and viscosity (**d**) data for GBM-3 aggregates generated in fibronectin-depleted medium supplemented with either 30 μg/ml or 300 μg/ml human fibronectin. Asterisks represent statistical significance by pair-wise Student *t*-test, *p* < 0.05. Note significant increase in stiffness and viscosity in response to increased concentrations of fibronectin

We first generated measurements of aggregate cohesion for GBM-1-4 treated in either DMSO or PD0325901, and confirmed that the cohesion measured was reflective of a true tissue surface tension (Table 1). We demonstrated that all GBM samples exhibited the defining characteristics of liquid-like behavior: (1) they display a constant surface tension when subjected to two different degrees of compression. Accordingly, the means of σ_1_ and σ_2_ when compared by a paired *t*-test are not significantly different, (2) the ratio of σ_2_/σ_1_ approaches 1 and is less than the ratio of the applied force at each successive compression (F_2_/F_1_). Table 1 shows that for all lines, a *t*-test comparing the ratios of σ_2_/σ_1_ to F_2_/F_1_ resulted in a *p* < 0.0001, indicating that the ratio of σ_2_/σ_1_ was significantly different than that of F_2_/F_1_, and 3) the surface tension of the aggregates is independent of aggregate volume. For the 4 GBM lines, combined aggregate volumes were plotted as a function of surface tension. Linear regression analysis yielded correlation coefficients, r^2^, of 0.031 and 0.071 for DMSO and PD0325901 treated aggregates, respectively, indicating that surface tension is independent of volume (Additional file 1: Figure S2). Figure 2a shows that PD0325901 treatment did not have an effect on aggregate surface tension. Surprisingly, however, generation of 3D spheroids in the presence of 300 μg/ml of serum fibronectin (sFn) resulted in a significant increase in aggregate cohesion (Fig. 2b), suggesting that the effects of PD0325901 may be through enhancement of α5β1 integrin-fibronectin interaction. Since actin is a fundamental mediator of cell and tissue mechanics, we reasoned that PD0325901 mediated changes in actin reorganization should result in a change in aggregate stiffness and viscosity. Interestingly, for aggregates generated in 30 μg/ml sFn (30 sFn), PD0325901 treatment slightly increased stiffness (Fig. 2c) but had no effect on viscosity (Fig. 2d). However, when aggregates were generated in the presence of 300 μg/ml sFn, both stiffness (Fig. 2c) and viscosity (2D) markedly increased. This suggests that fibronectin is an absolute requirement for PD0325901 to alter mechanical properties.

**Table 1:** Tissue surface tension measurements and confirmation of liquidity for DMSO-treated and PD0325901 treated aggregates of primary GBM cells

Line	σ_1_ dynes/cm ± s.e.m	σ_2_ dynes/cm ± s.e.m	σ_1,2_ dynes/cm ± s.e.m	*t*-test σ_1_ vs σ_2_ p	σ_2_/σ_1_	F_2_/F_1_	*t*-test σ_2_/σ_1_ vs F_2_/F_1_ p
GBM-1 DMSO	16.9 ± 1.9	19.4 ± 2.3	18.2 ± 1.5	0.4082	1.14 ± 0.05	1.36 ± 0.01	0.0003
GBM-1 PD03	18.4 ± 1.3	17.9 ± 1.2	18.1 ± 0.9	0.7951	0.98 ± 0.04	1.36 ± 0.02	<0.0001
GBM-2 DMSO	15.5 ± 1.4	16.9 ± 2.0	16.2 ± 1.2	0.5638	1.07 ± 0.08	1.35 ± 0.03	0.0072
GBM-2 PD03	15.2 ± 1.4	15.1 ± 1.0	15.2 ± 0.8	0.9035	1.02 ± 0.07	1.37 ± 0.04	0.0003
GBM-3 DMSO	8.5 ± 0.6	8.6 ± 0.7	8.6 ± 0.4	0.9041	1.01 ± 0.04	1.36 ± 0.02	<0.0001
GBM-3 PD03	8.9 ± 0.7	10.1 ± 0.6	9.5 ± 0.5	0.2389	1.15 ± 0.03	1.42 ± 0.07	0.0017
GBM-3 DMSO 300 FN	7.5 ± 0.7	7.1 ± 0.7	7.3 ± 0.5	0.6725	0.95 ± 0.03	1.28 ± 0.03	<0.0001
GBM-3 PD03 300 FN	32.7 ± 5.8	37.9 ± 6.6	35.3 ± 4.3	0.5550	1.17 ± 0.03	1.32 ± 0.03	0.0047
GBM-4 DMSO	16.2 ± 1.8	15.8 ± 1.2	16.0 ± 1.1	0.8833	1.01 ± 0.05	1.34 ± 0.01	<0.0001
GBM-4 PD03	20.6 ± 1.2	20.2 ± 1.0	20.4 ± 1.8	0.8312	1.02 ± 0.04	1.35 ± 0.02	<0.0001

For all cell lines, PD0325901 treatment did not result in a change in surface tension (pair-wise comparison by Student *t*-test, *p* > 0.05). GBM-3 was used to determine effects of exogenous fibronectin on surface tension. Here, addition of 300 μg/ml of soluble fibronectin resulted in a significant increase in aggregate surface tension (σ_1,2_) of 7.3 ± 0.5 to 35.3 ± 4.3 dynes/cm (pairwise Student *t*-test, *p* < 0.0001). Liquid behavior was confirmed by demonstrating that 1) surface tension measured at two different compressions, the second greater than the first, were not statistically different, and 2) that the ratio of σ_2_/σ_1_ approaches 1 and is less than the ratio of the applied force at each successive compression (F2/F1)

### PD0325901 increases resistance to shear-stress induced detachment, decreases cell motility and reduces dispersal velocity

**Fig. 3:**
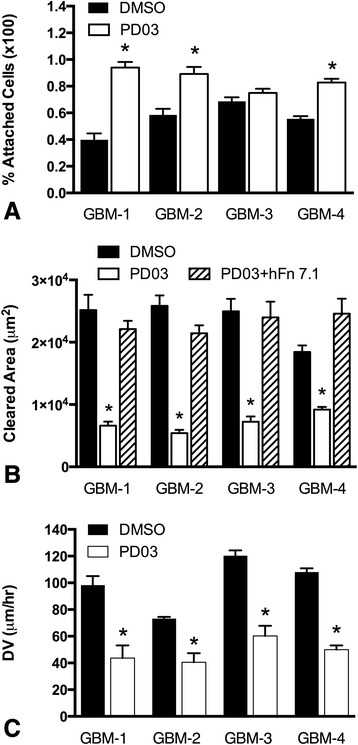
PD0325901 increases resistance to shear-stress induced detachment, decreases cell motility, and reduces aggregate dispersal velocity. Untreated and PD0325901-treated GBM cells attached to PET membranes were subjected to 30 dynes/cm of shear flow for 3 h, whereupon the number of cells retained on the membranes was quantified. For GBM-1, GBM-2 and GBM-4, PD0325901 treatment resulted in a significant retention of cells (**a**). A fluorescent microbead phagokinetic track assay was used to measure cell motility. For all GBM lines, PD0325901 significantly decreased cleared area. Co-incubation of cells with PD0325901 and 5 μg/ml anti-human fibronectin antibody, hFN 7.1, restored motility to control levels. Asterisks represent statistical difference using ANOVA, *p* < 0.0001, and Tukey’s multiple comparisons tests (**b**). The dispersal velocities of PD0325901-treated aggregates (*n* = 24) was significantly lower than those measured for PD0325901-treated aggregates (*n* = 24, **c**). For Fig. 3a and c, asterisks represent significant difference at *p* < 0.05 by pair-wise comparison using Student *t*-test

The effects of PD0325901 on cell shape, actin reorganization and aggregate viscoelasticity translate to significant changes in tumor cell behavior. Notably, PD0325901 treatment rendered GBM cells more resistant to shear-induced detachment (Fig. 3a), suggesting a stabilization of cell-ECM adhesion and a decrease in area cleared by motile cells in a phagokinetic microbead assay. For all lines, cleared area was reduced approximately 3-fold in response to PD0325901 treatment, indicating a significant decrease in cell motility. When experiments were performed in TCM containing 5 μg/ml mouse monoclonal anti-human fibronectin antibody, the motility of PD0325901-treated cells was restored to levels comparable to those of DMSO controls (Fig. 3b). This effect was not observed when a non-specific mouse IgG was used (Additional file 1: Figure S4). These results indicate that the principal mechanism of PD0325901-mediated decrease in motility is α5β1 integrin-fibronectin dependent. Collectively, the observed increase in attachment strength to substrate and decreased motility gave rise to a significant overall decrease in aggregate dispersal velocity. Figure 3c shows that spheroids of GBM cells differ in baseline dispersal velocities and that PD0325901 treatment reduces DV relative to DMSO controls.

### PD0325901 significantly alters pattern of dispersal and z-axis penetration

**Fig. 4:**
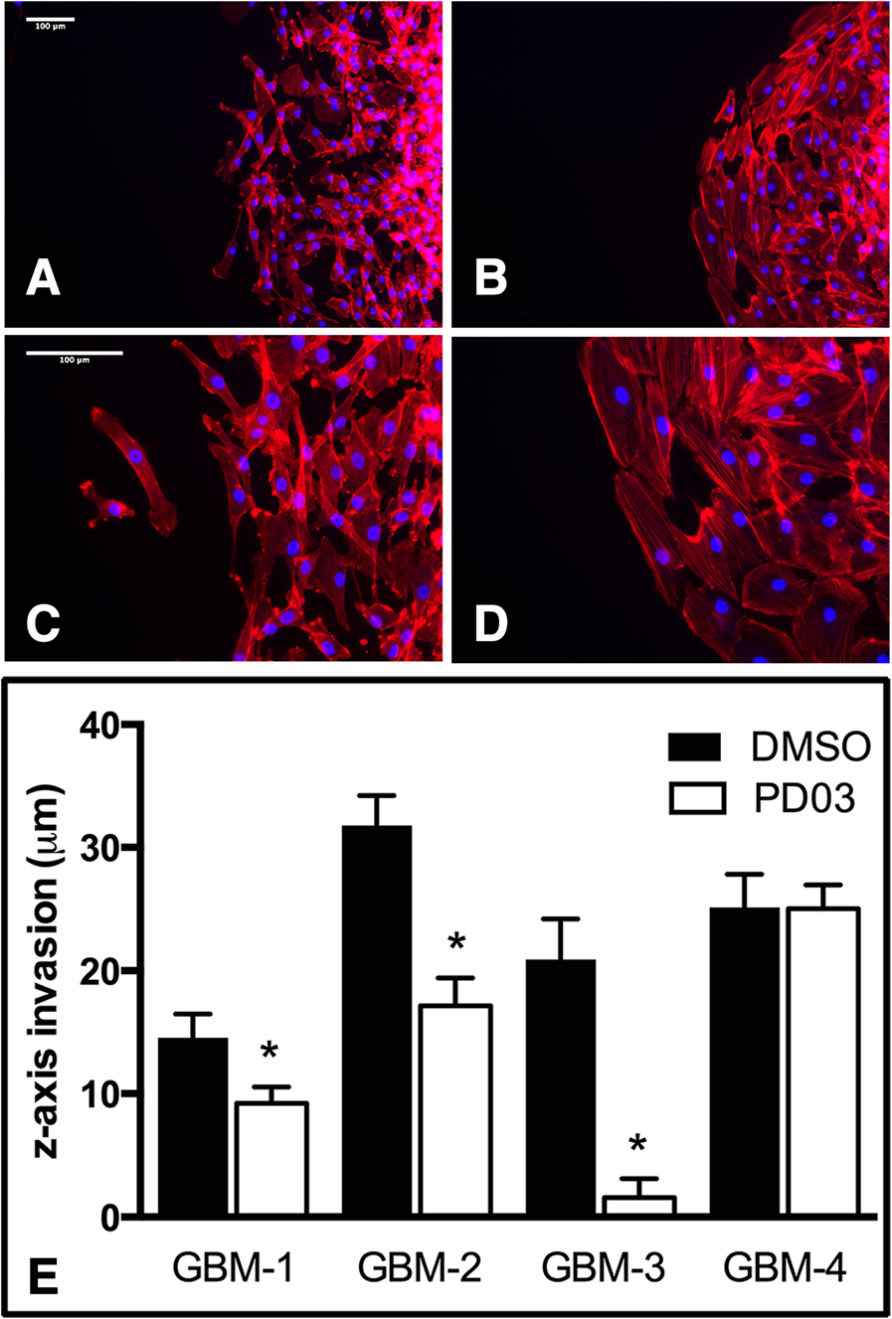
PD0325901 significantly reduces aggregate spreading and ex vivo dispersal. Aggregates of GBM-3 cells were plated onto tissue culture plastic in complete medium with DMSO (**a**, **c**) or PD0325901 (**b**, **d**) and incubated for 24 h, whereupon they were fixed, permeabilized and stained with rhodamine-phalloidin. Low Mag (**a**, **b**) and High Mag (**c**, **d**) images were collected. Note single cell dispersal from untreated aggregates (**a**, **c**), in contrast to a higher level of cell-cell contact and actin stress fibers in response to PD0325901 treatment. (**b**, **d**). Scale bars in (**a**) and (**c**) are 100 μm. For GBM-1-3, PD0325901 decreased z-axis dispersal of GBM cells through a normal human astrocyte seeded 3D scaffold (**e**). Asterisks represent pair-wise comparison, Student *t*-test *p* < 0.05. No significant difference in z-axis dispersal was observed for GBM-4 (*p* = 0.9731)

Treatment with the MEK inhibitor also resulted in a change in the pattern of dispersal. Whereas, the advancing edge of DMSO-treated aggregates dispersed as single cells (Fig. 4a, c), the leading edge of PD0325901-treated aggregates advanced as a sheet (Fig. 4b, d). Moreover, actin in advancing cells of DMSO-treated aggregates appeared to be cortical (Fig. 4b), whereas in treated aggregates, actin was arranged in stress fibers (Fig. 4d). This change in spreading behavior is likely associated with reduced cell motility, causing cells escaping the aggregate mass to accumulate behind the advancing front. PD0325901 treatment also resulted in a significant reduction in z-axis dispersal for three of the four lines (Fig. 4e). The z-axis dispersal distance of PD0325901-treated GBM-1 and GBM-2 cells was approximately 2-fold less than that of the vehicle controls. GBM-3 cells responded more actively, their z-axis dispersal distance becoming reduced approximately 13-fold relative to controls.

### PD0325901 reduces growth rates of conventional 2D cultures and 3D spheroids of GBM cells

**Fig. 5:**
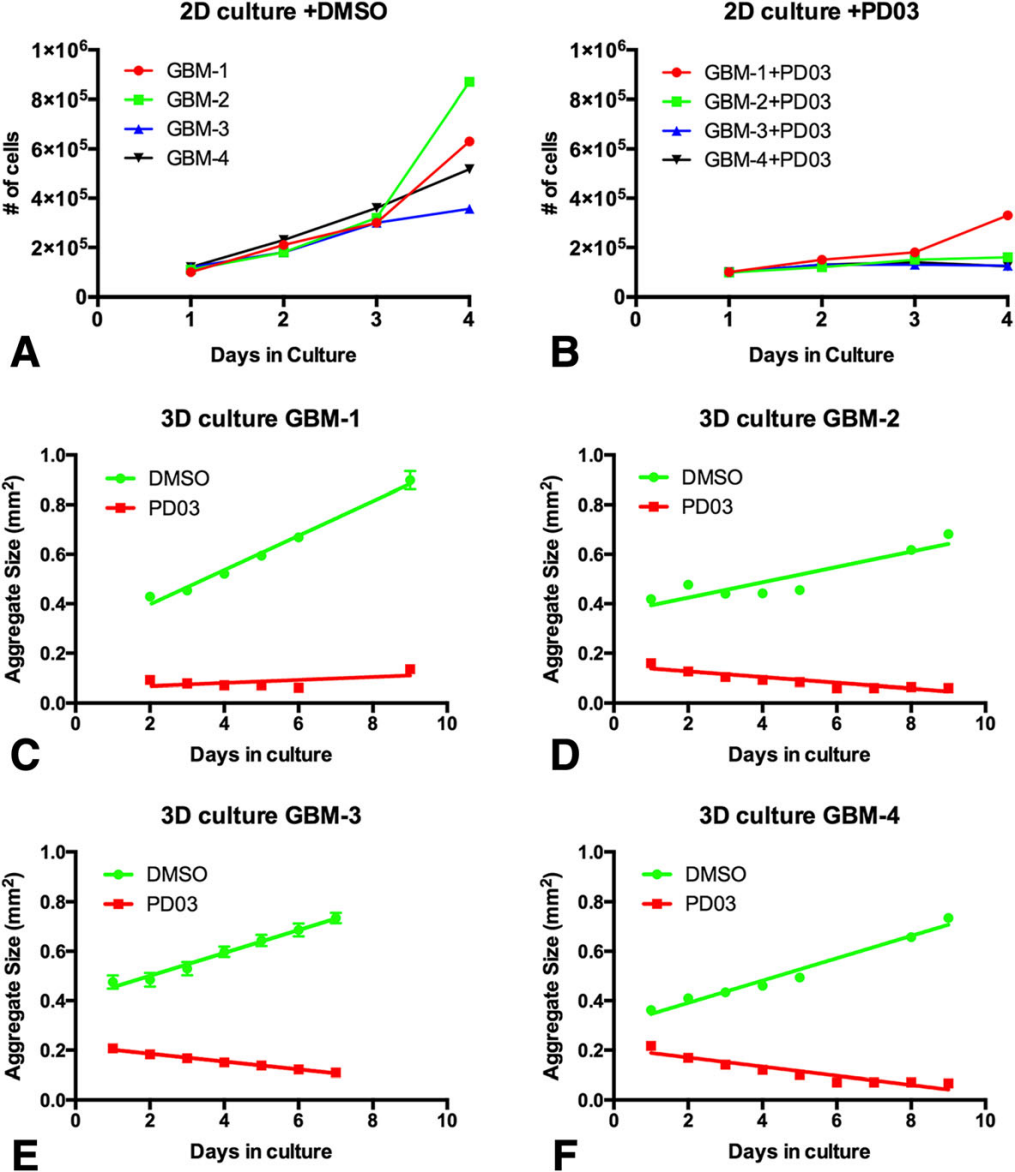
PD0325901 reduces growth rates of conventional 2D cultures and 3D spheroids of GBM cells. Growth rate for conventional 2D cultures of the GBM lines was measured either in DMSO (**a**) or PD0325901 (**b**). Fifty-thousand cells were plated and proliferation was monitored over a 4-day period. PD0325901 treatment significantly reduced the growth rate of 2D cultures. Aggregates (*n* = 6 for each line and treatment) of GBM-1 (**c**), GBM-2 (**d**), GBM-3 (**e**) and GBM-4 (**f**) were cultured either in the absence or presence of PD0325901 and area was measured for each aggregate once/day for 9 days. Linear regression was used to analyze the data. Only regression lines with an r^2^ of 0.95 or higher were used. Regression lines depicted are average area as a function of time. Growth rate was significantly reduced by PD0325901 for all GBM lines as demonstrated by a significant shallowing of the slope of the line (ANCOVA, *p* < 0.0001). For GBM-1-4, growth rate was reduced 11.6, 2.7, 2.9, and 2.5-fold, respectively

Previous studies demonstrated that the growth rate of various immortalized GBM cell lines was markedly reduced by PD0325901 [20]. We tested our primary GBM lines to determine whether treatment had a similar effect when cells were grown as conventional 2D cultures and as spheroids. Figure 5 (a, b) shows that PD0325901 significantly decreases the growth rate of conventional 2D cultures of GBM-1-4 as compared to DMSO controls. We then repeated the experiment using spheroids of GBM cells. Figure 5c-f shows that PD0325901 treatment significantly reduced the growth rate of GBM aggregates since the slope of the growth curves was significantly reduced by treatment relative to that of controls. Linear regression analysis of the 3D growth curves revealed a 2–11 fold reduction in growth rate when spheroids of GBM cells were treated with the drug. In fact, PD0325901 treated aggregates appeared to decrease in size over time. This suggests that cells are either dying and sloughing off the surface of the aggregate – this was not observed for aggregates incubated for 2 days (Additional file 1: Figure S3, panel a) or 4 days (Additional file 1: Figure S3, panel b) on agarose plates – or that aggregates became more compact over time. This appears to be the case inasmuch as PD0325901 treatment resulted in more compact aggregates (Additional file 1: Figure S3, Panel c). It is likely that the observed compaction was due to overall contraction of cell size.

## Discussion

Therapies aimed at containing tumor cell dispersal could provide a powerful path towards extending the time of disease-free and overall survival of glioblastoma patients. Identifying drugs that can target molecular pathways involved in dispersal would provide valuable insight towards this goal. Our previous studies showed that Dexamethasone, an FDA approved drug to treat tumor-related edema in GBM, can also decrease in vitro and ex vivo dispersal of primary human GBM cells. It does so by activating α5β1 integrin and subsequent restoration of FNMA and re-organization of cortical actin into stress fibers. In turn, these changes engender an increase in the strength of intercellular cohesion, increased attachment of tumor cells to substrate, and reduced cell motility. The net effect is an overall reduction in dispersal [1, 27]. The effects of Dex, however, are pleiotropic and the drug likely targets many pathways, which in part may explain the many side-effects associated with Dex treatment. Identifying drugs that are more specific in their targeting of dispersal-related pathways is therefore important.

In this study, we explore whether inhibition of the MAPK/ERK pathway, a critical regulator of processes underlying invasion and metastasis [28], could have similar effects on GBM dispersal. We tested the effects of the MEK inhibitor, PD0325901, on 4 primary GBM cell lines that were previously used to assess the effects of Dex on dispersal [1, 21]. Studies have shown that certain GBM lines do not respond to MEK inhibitors [20]. We therefore assessed whether our lines are responsive to PD0325901 by determining whether treatment results in a decrease in the levels of phospho-ERK. All 4 lines responded to the drug. We previously established that the cell lines were all deficient in their capacity for FNMA [1]. In contrast to Dex, treatment with PD0325901 did not result in a significant increase in FNMA. However, treatment with the MEK inhibitor resulted in a remarkable change in cell shape and in the reorganization of actin from cortical into stress fibers. This was particularly evident when actin was visualized in 3D spheroids. Given that the actin cytoskeleton is a fundamental mediator of cell and tissue stiffness [29], we posited that a shift in actin organization would correspond to a change in tissue stiffness.

Stiffening of the ECM is considered to be a hallmark of fibrotic lesions and has been demonstrated to modulate cell invasion and migration [30]. The current study focused on whether aggregate stiffness and viscosity could modulate dispersal. We quantified stiffness and viscosity using methods based on ellipsoid relaxation, specifically after the deforming external force is removed [31, 32]. The aggregate was modeled as a Kelvin-Voigt viscoelastic body [33, 34]. Unexpectedly, PD0325901 treatment only resulted in a modest increase in aggregate stiffness but not of viscosity. However, when aggregates were generated in higher concentrations of fibronectin, both stiffness and viscosity increased significantly. This is important for several reasons. First, the fibronectin gene has been shown to be up-regulated in GBM [35]. Accordingly, tumors able to respond to PD0325901 and in the presence of high concentrations of fibronectin, could, in principle, become stiffer and more viscous. Stiffer tumors have previously been shown to be less invasive and to grow more slowly [36]. Few studies have addressed the issue of tumor viscosity and those that have focus on applications of magnetic resonance elastography in liver tumors where fibrosis is a key parameter. In those studies, tumor viscosity appeared to be higher in malignant tumors [37]. In GBM, however, fibrosis is not typically observed. In GBM spheroids, the increase in viscosity in response to PD0325901 treatment was likely due to higher binding energy between the activated α5β1 integrin and fibronectin. This would effectively increase the friction between cells and the ECM. This increase in friction could significantly reduce the capacity for dispersal of tumor cells from the primary mass.

Treatment also resulted in the localization of p-FAK at sites of cell-substrate attachment. This is consistent with the observed resistance to flow-induced substrate detachment of GBM cells, and to decreased motility. Since cells require intermediate levels of cell-ECM adhesion to be optimally motile [38], an increase in the strength of cell-ECM adhesion past this point might stabilize adhesion to substrate to a point that significantly reduces cell movement, and consequently, dispersal. Decreased motility also appears to be associated with a significant decrease in dispersal velocity of GBM aggregates. Since PD0325901 treatment did not restore FNMA, it is likely that decreased motility rather than increased cohesion is the physical mechanism that restrains the detachment of tumor cells from the mass. Indeed, cells at the leading edge of treated aggregates appear to attach tightly to substrate causing cells behind them to pile up, again pointing to reduced motility as the primary restraint for detachment. For three of the four primary GBM lines, PD0325901 also significantly reduced the ability of single GBM cells to disperse through an astrocyte-seeded scaffold. It is not possible to differentiate between the effects of PD0325901 on decreased motility and ability to disperse through the scaffold, however, it is possible that on a single cell level, the re-organization of actin into stress fibers may have effectively rendered cells less compliant and inhibited their capacity to sufficiently deform and squeeze through pores established by the physical environment established by the scaffold. It is important to note that for GBM-4, treatment did not reduce z-axis dispersal. It is possible that in this line, compliance was not effected by treatment, thus allowing cells to penetrate into the scaffold.

Lastly, MEK inhibitor treatment also appears to significantly reduce growth rate of these primary GBM lines in both conventional 2D and in 3D cultures. Other studies have demonstrated in vivo efficacy of PD0325901 in reducing tumor growth in preclinical orthotopic models of glioblastoma [18]. Our study provides compelling evidence that PD0325901 can also reduce dispersal. Growth and dispersal contribute significantly to recurrence. Accordingly, the drug has the potential to significantly delay the onset of recurrence in GBM.

Identifying agents that can contain the primary or recurrent tumor could significantly improve targeted delivery of chemotherapeutic agents and increase the likelihood of total surgical resection. We have previously identified Dexamethasone (Dex) as a potential candidate to reduce dispersal of GBM [1]. Interestingly, the doses required to elicit a dispersal inhibitory response are significantly lower than those typically used to reduce edema [1]. Clinically, MEK inhibitors are generally well tolerated. Commonly occurring toxicities include rash, diarrhea, fatigue, peripheral oedema and acneiform dermatitis. Life-threatening toxicities associated with MEKi are extremely rare. Long-term use is possible providing that adverse events are monitored and dose or treatment schedules are modified, as required [39]. The measureable outcome for MEK inhibitor studies focus on their ability to reduce tumor size. Here, we show an added benefit of one MEK inhibitor as a potential deterrent of tumor cell dispersal. Whereas Dexamethasone readily crosses the blood–brain barrier, some MEK inhibitors, including trametinib, have demonstrated limited brain distribution due to association with the P-glycoprotein efflux transporters found at the blood–brain barrier [19]. Perhaps a strategy in which MEK inhibitors are used as interstitial chemotherapy, followed by continued administration of low-dose Dex, could significantly improve prognosis of this devastating disease.

## Conclusions

This study demonstrates that it is possible to impede dispersal of GBM by inhibiting the MAPK/ERK pathway using the MEK inhibitor PD0325901. To our knowledge, this is the first demonstration that the drug can also impede GBM dispersal. Containing the primary or recurrent tumor by interstitial administration of MEK inhibitors could significantly improve delivery of chemotherapeutic agents and increase the likelihood of total surgical resection. This could significantly extend the time of disease-free and overall survival of glioblastoma patients.

## Additional file

Additional file 1: Analytical method for measurement of aggregate viscoelasticity. (DOCX 58681 kb)

## Abbreviations

AGM: Astrocyte growth medium
Dex: Dexamethasone
DMSO: dimethyl sulfoxide
ECL: Enhanced chemiluminescence
ECM: Extracellular matrix
EMCC: Electron multiplying charge coupled device
EMEM: Eagle’s Minimal Essential Medium
FCS: Fetal calf serum
FNMA: Fibronectin matrix assembly
GBM: Glioblastoma multiforme
GLA: Gamma-linolenic acid
MAPK/ERK: mitogen activated protein kinase/extracellular signal regulated kinase
NHA: Normal human astrocytes
PAGE: polyacrylamide gel electrophoresis
PBS: Phosphate buffered saline
PVDF: polyvinylidine fluoride
SDS: sodium dodecyl sulphate
sFn: Serum fibronectin
TST: Tissue surface tensiometry
