# Supplementary material for: Inhibition of glioblastoma dispersal by the MEK inhibitor PD0325901
Source: BMC Cancer. 2017 Feb 10;17:121. doi: 10.1186/s12885-017-3107-x (PMC5303286; doi:10.1186/s12885-017-3107-x)
Supplement: Additional file 1: — Analytical method for measurement of aggregate viscoelasticity. (DOCX 58681 kb) [file 12885_2017_3107_MOESM1_ESM.docx]

## Additional file

### Theory and data analysis

The cell aggregate is modeled as a linear viscoelastic solid bulk encapsulated by an interface with a constant surface tension. A continuum Kelvin-Voigt model is used to characterize the bulk viscoelasticity with the following constitutive relation:

$\mathbf{T}=-p\mathbf{I}+\mu_{b}\left[ \nabla\mathbf{u}+\nabla\mathbf{u}^{T} \right]+\eta\left[ \nabla\dot{\mathbf{u}}+\nabla{\dot{\mathbf{u}}}^{T} \right]$.

Here **T** is the stress tensor, **u** is the displacement vector, *p* is a pressure field resulting from incompressibility of the material (isovolumetric process), and **I** is the unit tensor. μ_b_ and η are bulk elastic modulus and viscosity, respectively. In this model, the total stress is the sum from both the elastic and viscous responses. Stress balance leads to the divergence free condition,

$\nabla\cdot\mathbf{T}=0$.

In addition, incompressibility dictates

$\nabla\cdot\mathbf{u}=0$.

These form the governing equations for the displacement and velocity fields. Boundary conditions are prescribed at the interface where force is balanced in both the normal and tangential directions; the surface tension only acts in the normal direction.

The physical problem defined above can be solved analytically if the displacement **u** is small in magnitude compared to the radius of aggregate. In the quasi-spherical regime, the solution to the system can be linearly decomposed for each spherical harmonic mode:

$$r\left( \Theta,t \right)=R\left[ 1+f_{2,0}\left( t \right)Y_{2,0}\left( \Theta\right)+f_{3,0}\left( t \right)Y_{3,0}\left( \Theta\right)+\cdots\right].$$

Here *r*(Θ,*t*) defines the instantaneous aggregate shape. *R* is the radius of a sphere of equivalent volume. *Y_l,m_* is a spherical harmonics of the (*l*,*m*) mode; *f_l,m_* is the corresponding magnitude. The mode (1,0) is not included as it corresponds to translation. A solution can be obtained for each mode, yet a contour analysis from our experiments indicates that *f_2,0_* is the dominant mode (Fig. S1G) and the focus of our analysis. A double-exponential relaxation behavior is revealed:

ε$\left( t \right)=Ae^{-t/\tau_{L}}+Be^{-t/\tau_{S}}$, (S1)

where

$\tau_{L}=\left( \frac{\mu_{b}}{\eta} \right)^{-1}$, $\tau_{S}=\left( \frac{\mu_{b}}{\eta}+\frac{20}{19}\frac{\sigma}{\eta R} \right)^{-1}$. (S2)

Here, the shape factor, ε, is related to *f_2,0_* via a simple scaling factor, ε $=\sqrt{\frac{5}{4\pi}}f_{2,0}$. For a shape close to ellipsoid, ε $\approx\frac{2}{3}\left( \frac{a}{b}-1 \right)$ or ε $\approx\frac{4}{3}\frac{a-b}{a+b}$ to the leading order, where *a* and *b* are the lengths of the semi- axes. The detailed solution process is found elsewhere [1].

Figs. S1A-F show representative images of a GBM aggregate in different stages of deformation and shape relaxation. An in-house edge detection code was written in MATLAB to accurately capture the edge contour of the aggregate in each frame, as marked by blue in Fig. S1A. Fourier-Legendre analysis of each contour yields the decomposed mode magnitudes *f_l,m_*. Fig. S1G shows mode evolution for an exemplary case, confirming the dominance of *f_2,0_*, which is equivalent to a spheroidal shape to leading order. Fig. S1H shows the relaxation process in terms of ε. A double exponential fitting according to Eq. (S1, S2) is shown in solid on top of the data; the R­^2^ is 0.998, demonstrating an excellent agreement. The fitting process reveals two time scales, τ*_L_* and τ*_S_*. Per Eq. (S2) and knowing the surface tension, σ, from an independent measurement outlined in the proper text, we can extract the bulk viscoelastic properties, namely, μ_b_ and η. The results are presented in Fig. 2 of the proper text, where stiffness is μ_b_, and viscosity is η.

Assuming axisymmetry, we also conduct surface area and volume analysis during shape relaxation for each experiment. Figs. S1I-J show exemplary plots of surface area and volume as a function of time, respectively. It can be observed that the surface area decreases as the aggregate shape recovers toward sphericity. The volume, on the other hand, remains approximately constant within the uncertainty of the experimental setup and edge detection algorithm. This further verifies our hypothesis of isovolumetric relaxation and liquid-like behavior of the surface.

[1] Liu, L, M. Yu, H. Lin and R.A. Foty. (2017). Deformation and relaxation of an incompressible viscoelastic body with surface viscoelasticity. J. Mechanics and Physics of solids 98:309-329.


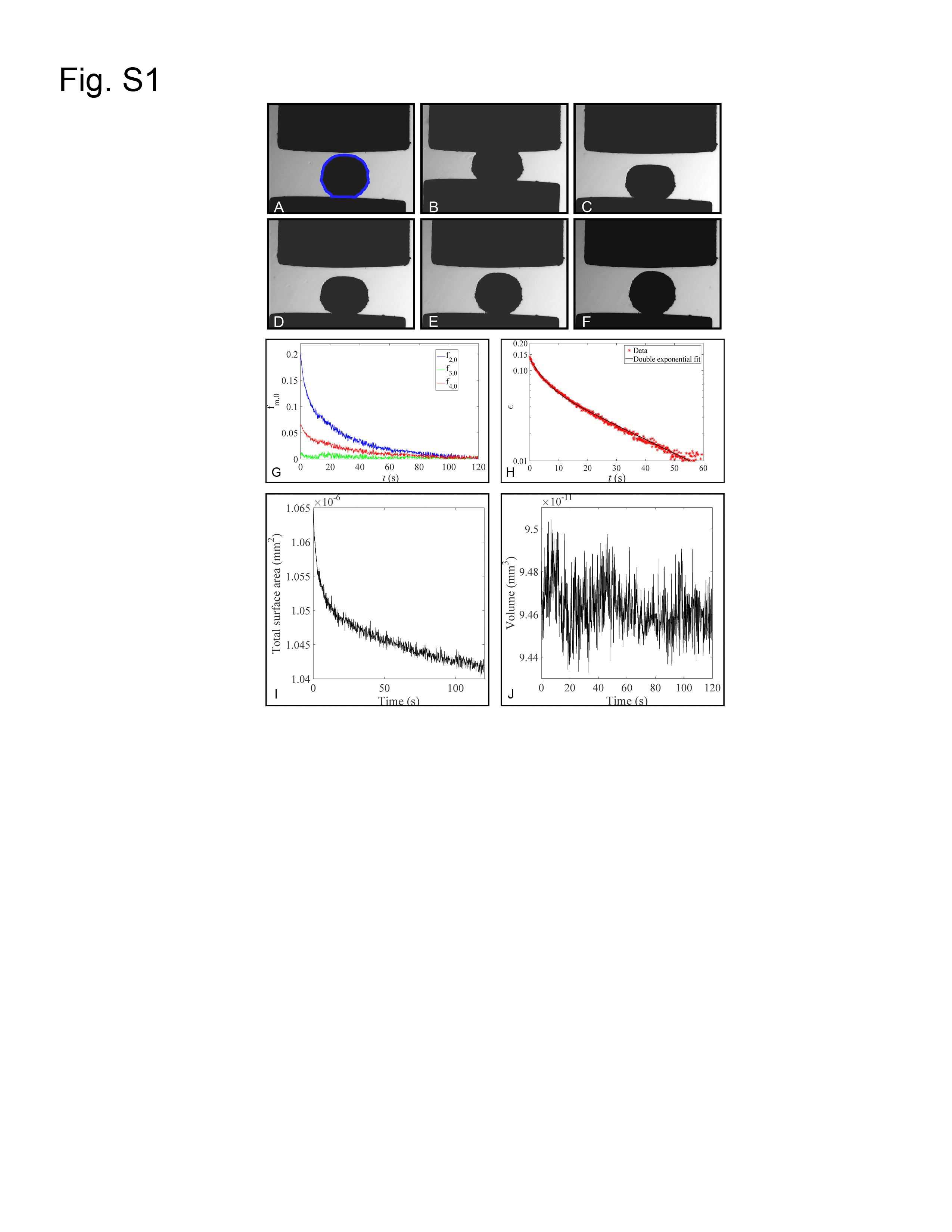


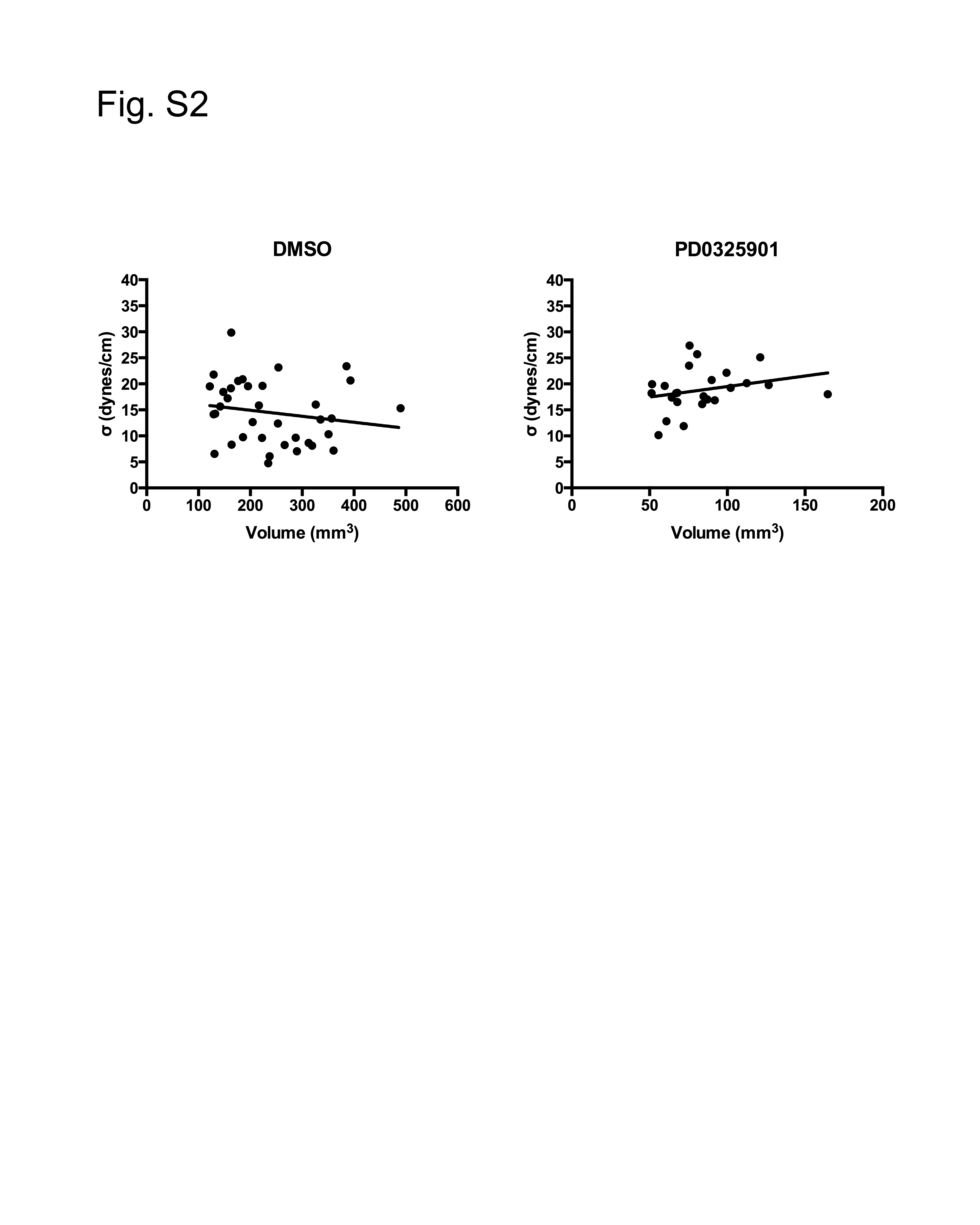


Fig. S3


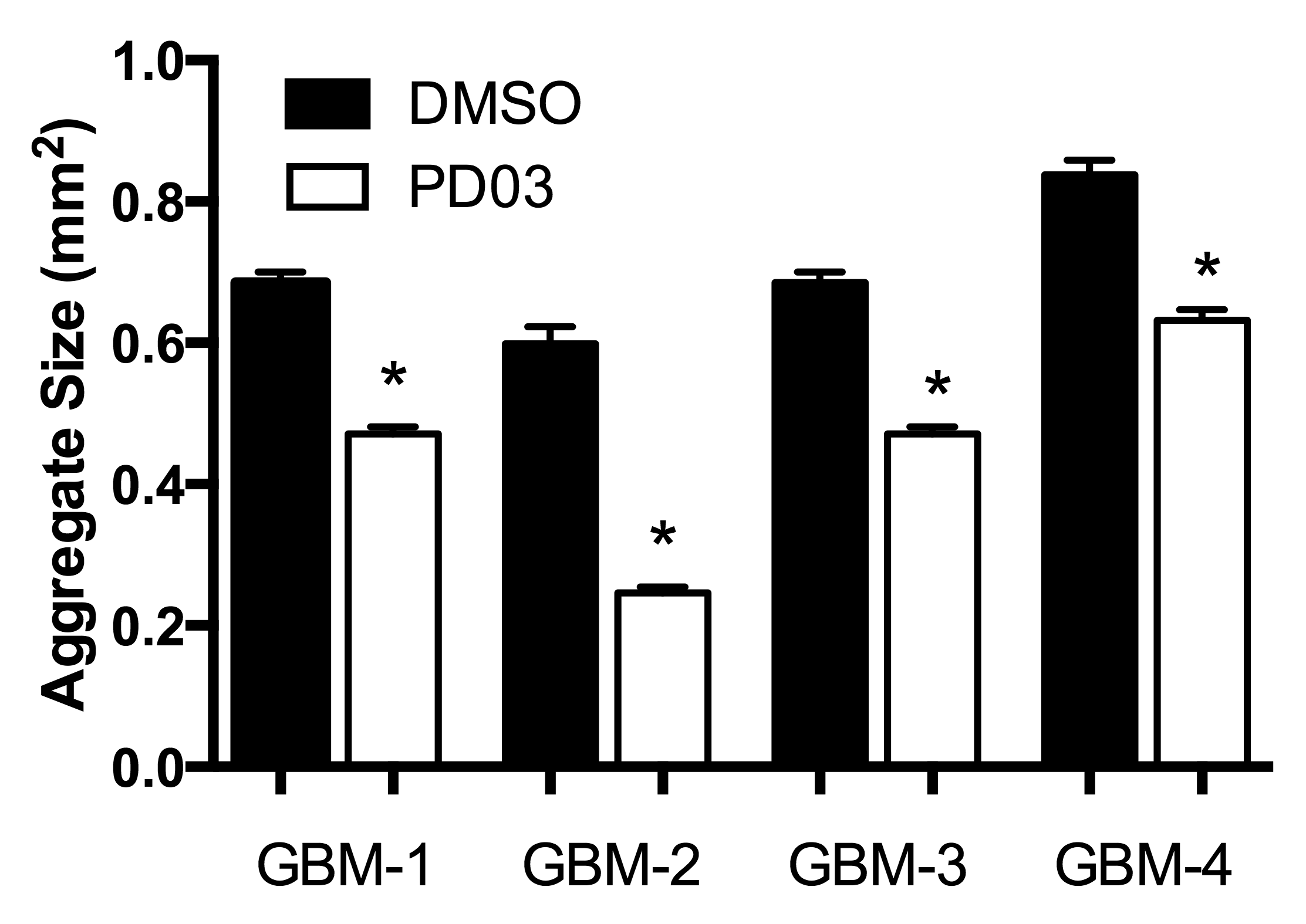


A

B

C


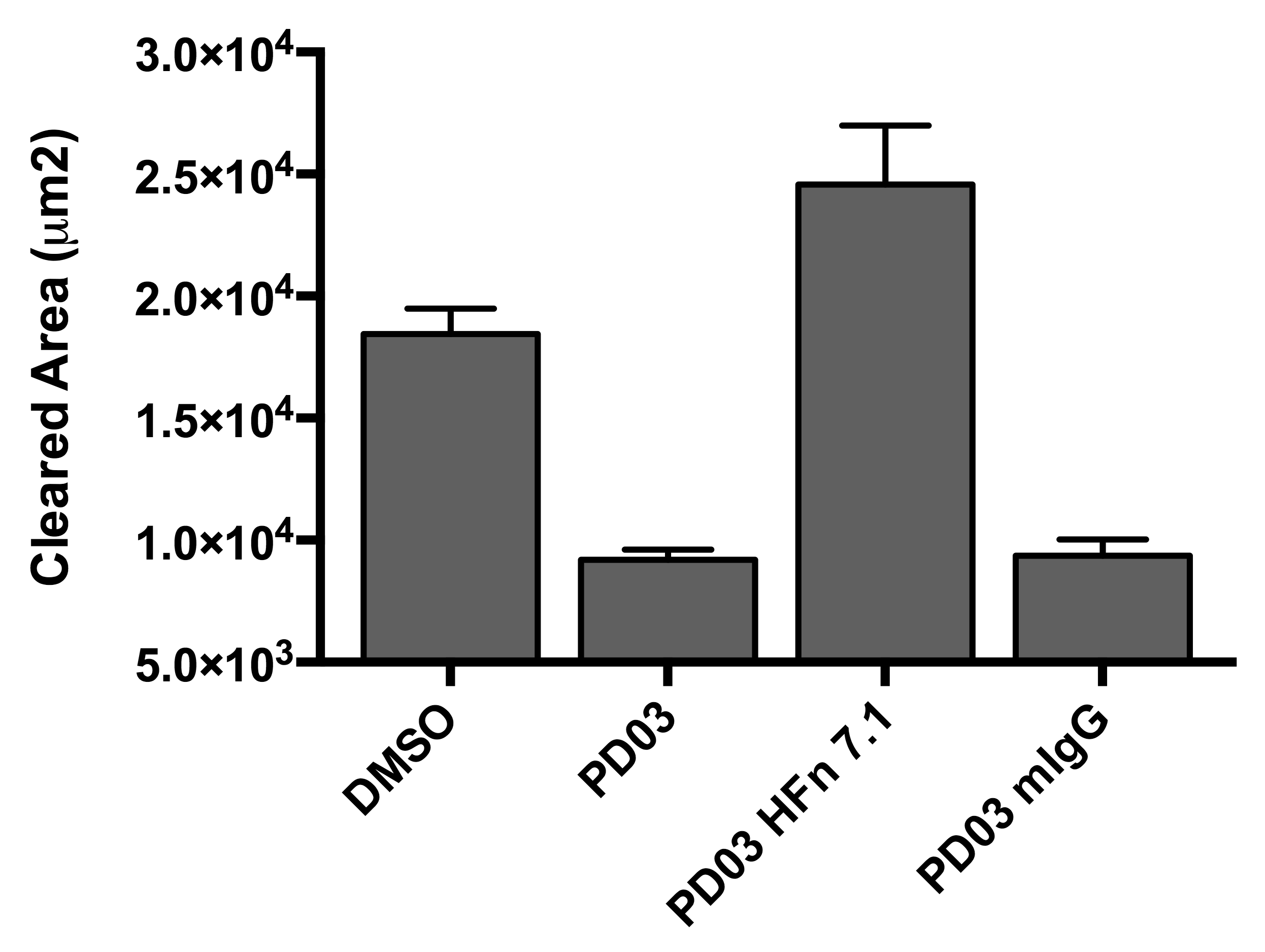


Fig. S4

**Supplemental Figure Captions**

**Fig. S1.** Representative images of aggregate deformation and relaxation. (A) pre-compression image with an overlay of the edge contour (blue) as detected by an in-house MATLAB code. (B) aggregate deformed by compression. (C-F), aggregate relaxation to original contour. (G) Plots of three mode shapes as a function of time during aggregate shape relaxation. The plot clearly demonstrates that is the dominant mode. (H), an exemplary fit of shape relaxation data describing a double exponential with a coefficient of determination r^2^ of 0.998. Plots of surface area (I) and volume (J) calculated from code-detected contour assuming axisymmetry. The surface area decreases as aggregate shape relaxes towards sphericity, whereas the volume appears to remain constant.

**Fig. S2.** Surface tension as a function of volume for aggregates of GBM-1-4. For the 4-GBM lines, combined aggregate volumes were plotted as a function of surface tension. Regression analysis generated a correlation coefficient, r^2^=0.031 for DMSO-treated aggregates, and r^2^=0.071 for PD0325901-treated aggregates. The slopes of the regression lines did not significantly deviate from zero (p=0.300 and p=0.071 for DMSO and PD0325901, respectively), indicating no relationship between surface tension and volume.

**Fig. S3.** PD0325901 treatment induces contraction of GBM aggregates independent of cell death. Untreated (UT), DMSO treated, and PD0325901 treated aggregates of GBM-2 were cultured for (A) 2 days, or (B) 4 days on agarose-coated plates whereupon they were stained with propidium iodide. Brightfield and fluorescence images were captured. Note that the significant decrease in aggregate size in response to PD0325901 treatment was independent of any difference in surface cells stained with propidium iodide. (C) To measure aggregate compaction, cells were re-suspended at a concentration of 2.5x10^6^ cells/ml in TCM and incubated for 24 hours either in DMSO or in 1μm PD0325901. Cells coalescing at the bottom of the drops formed sheets. After 24 hours in culture, images of the sheets were captured and digitized. Image analysis was performed using iVision imaging software. Image contrast was optimized and outlines were automatically traced. The number of pixels within the outlined image was calculated by the imaging software. Data points representing the mean and standard error for aggregate surface area expressed in mm^2^ was calculated from 10-15 aggregates of GBM each cell line. Note the significant decrease in aggregate size in response to drug treatment. * Represent significant difference at p<0.05 by pair-wise Student t-test.

**Fig. S4.** Motility assay of GBM-4 cells treated with PD0325901 and either hFn 7.1 mouse monoclonal anti-human fibronectin antibody or non-specific IgG control. As evident in Fig. S4, treatment with PD0325901 significantly reduced cleared area, whereas addition of hFn 7.1 restored motility to levels similar to those of the DMSO controls. A non-specific IgG control failed to restore motility.
